# Risk factors for *Candida parapsilosis* bloodstream infection in a neonatal intensive care unit: a case-control study

**DOI:** 10.1186/s13052-017-0332-5

**Published:** 2017-01-19

**Authors:** Carmine Garzillo, Maria Bagattini, Lidija Bogdanović, Anna Di Popolo, Vita Dora Iula, Maria Rosaria Catania, Francesco Raimondi, Maria Triassi, Raffaele Zarrilli

**Affiliations:** 1 Department of Public Health, University of Naples “Federico II”, Via S. Pansini n.5, 80131 Naples, Italy; 2 Department of Molecular Medicine and Medical Biotechnologies, University of Naples “Federico II”, Naples, Italy; 3 Division of Neonatology, Department of Medical Translational Sciences, University of Naples “Federico II”, Naples, Italy

**Keywords:** *Candida parapsilosis*, Neonatal intensive care unit, Healthcare-associated infections, Risk factor analysis, Low birth weight

## Abstract

**Background:**

*Candida parapsilosis* is increasingly responsible for invasive candidiasis in neonates. This study investigates phenotypic and genotypic features of *C. parapsilosis* microbial isolates and underlying clinical conditions associated with acquisition of *C. parapsilosis* in a neonatal intensive care unit (NICU) in Italy.

**Methods:**

Identification of *C. parapsilosis* was performed by VITEK® 2 and MALDI TOF and confirmed by analysis of internal transcribed spacer ribosomal DNA sequences. Genotyping was performed by PCR fingerprinting. Antifungal susceptibility of strains was evaluated by microdilution. A case-control study was designed to identify risk factors for *C. parapsilosis* bloodstream infection.

**Results:**

During the study period (April 2009- April 2012), *C. parapsilosis* was responsible for 6 umbilical catheter and 11 central catheter-associated bloodstream infection in 17 neonates in the NICU. Molecular typing identified identical fingerprinting profile in all *C. parapsilosis* isolates from neonates. Fifteen of 17 *C. parapsilosis* isolates were susceptible to all antifungal drugs, two isolates were resistant to fluconazole and intermediate susceptible to itraconazole. Low birthweight, gestational age and time to exposure to assisted ventilation were risk factors for *C. parapsilosis* infection in neonates in the NICU at univariate and multivariate analysis.

**Conclusion:**

*C. parapsilosis* bloodstream infections in the NICU were caused by a single epidemic clone. Low birthweight, gestational age and time to exposure to invasive devices, with predominance of assisted ventilation, were the clinical conditions associated with *C. parapsilosis* bloodstream infection in the NICU.

**Electronic supplementary material:**

The online version of this article (doi:10.1186/s13052-017-0332-5) contains supplementary material, which is available to authorized users.

## Background

Healthcare-associated infections (HAI) are frequent complications occurring during hospitalization of neonates in intensive care units, resulting in increased morbidity and mortality, prolonged lengths of stay, and increased medical costs [1, 2]. Low-birth weight and use of central venous catheterization and mechanical ventilation were identified as risk factors of HAIs in the neonatal intensive care unit (NICU) [3–6]. Etiology of device-associated infections in NICUs shows that *Candida spp*. are among the most frequent pathogens responsible for HAIs in NICUs, followed by gram positive or gram negative bacteria according to different clinical setting [1–9]. Although *Candida albicans* remains the most frequently isolated in many centers [9, 10], *Candida parapsilosis* has emerged as the most frequent non-albicans *Candida* species and the predominant pathogen of invasive candidiasis in neonates [8, 11–17]. In the tertiary care NICU of University Hospital in Naples, Italy, *C. parapsilosis* was the most frequent pathogen responsible for device-associated bloodstream infections (BSI) during 2006–2010 [6].

Aims of the present study were to: i) analyse phenotypic and genotypic features of *C. parapsilosis* clinical isolates from the NICU of University Hospital in Naples, Italy; ii) identify underlying clinical conditions associated with *C. parapsilosis* bloodstream infection in the NICU.

## Methods

### Setting

The University of Naples “Federico II” NICU is a tertiary care level NICU with a total of 25 incubators and cradles. The ward serves the University Obstetric Clinic (approximately 2000 births/year) which is both a high risk pregnancy center and an obstetric emergency service. Moreover, the NICU admits outborn neonates from the regional Newborn Emergency Transport Service. The tertiary-level NICU of the Federico II University Hospital in Naples has approximately 350 admissions per year. Fluconazole prophylaxis for neonates ≤1500 g BW at dose of 6 mg/kg body weight every 72 h during the first two weeks and then every 48 h [18] is active at the NICU of Federico II University Hospital.

### Surveillance of healthcare-associated infections

HAIs active, patient based surveillance on neonates with >2 days NICU stay was performed as previously described [6]. Data regarding birth (birth weight (BW), gestational age, type of delivery, and Apgar score), invasive device exposure (days of umbilical and central line catheterization and of invasive ventilation) and infections were collected. HAIs were defined using standard Centers for Disease Control and Prevention definitions adapted to neonatal pathology and were considered to be healthcare-associated if they develop >2 days after NICU admission [19–21]. Central line-associated BSI and umbilical catheter-associated BSI were attributed if a central line and an umbilical catheter, respectively, were in place at the time of or within 48 h before infection onset [21]. Surveillance swabs from the nose, pharynx and rectum of each neonate admitted to the ward were analyzed weekly as part of a screening programme for surveillance of HAIs. Environmental cultures (surfaces, including walls, floor, beds and the drug trolley, handwashing sinks, disinfectants, equipment and staff hands) were obtained using a brain —heart broth moistened cotton swab during four environmental microbiological investigations.

### Mycotic strains and microbiological methods

All *C. parapsilosis* clinical isolates from blood cultures and from surveillance swabs of the patients hospitalized in the NICU and *C. parapsilosis* environmental isolates from the NICU between April 2009 and April 2012 were included in the study. All the strains were grown on Sabouraud with CAF 50 mg/ml (Oxoid Basingstoke, UK) and were identified using standard procedures i.e. morphology on Sabourad with CAF 50 plate, determination of the colonies color non *Candida* agar chromogenic medium, microscopic examination and biochemical analysis by VITEK® 2 automatic system (bioMèrieux Marcy-L’Etoile France) and by MALDI TOF mass spectrometry as previously described [22]. All strains were stored at −80 °C in glycerol solution.

Identification of *C. parapsilosis* group was confirmed by PCR amplification and analysis of internal transcribed spacer ribosomal DNA sequences [23]. Genotyping of microbial isolates was performed by PCR fingerprinting as previously described [24]. In brief, fingerprinting profiles were determined by scoring bands from GACA4, M13, T3B and OPA-03 PCR single primers; at least 22 digits were considered for each isolate.

### Antimycotic susceptibility testing

Susceptibility to amphotericin B, caspofungin, fluconazole, itraconazole, voriconazole, posaconazole, anidulafungin, micafungin, 5-fluorocytosine was analyzed using the colorimetric microdilution test Sensititre (Trek Dignostic Systems, Ltd, East Grinstead, Sussex, UK). After overnight growth on Sabouraud dextrose agar at 35 °C, appropriate dilutions of each strain were prepared according to manufacturer’s recommendations. The Panels for the Sensititre test were inoculated and then incubated at 35 °C in a non-CO_2_ incubator. The positive growth well was examined after 24 h incubation. If no growth was detected, the panel was incubated for a further 24 h. MICs were determined by visual inspection of the plates; the concentration of the first well that remained blue (absence of growth) was recorded as the MIC. *C. krusei* ATCC 6258 and *C. parapsilosis* ATCC 22019 were used as quality control isolates. The results of the in vitro susceptibility tests were interpreted according to the established CLSI MIC break-points [25].

### Study design

A retrospective case-control study was designed to identify individual risk factors for *C. parapsilosis* bloodstream infection in the NICU. Patients enrolled in the analysis were all neonates who were in the NICU during the study period (April 2009–April 2012) for at least 48 h and were divided into 10 groups. Cases were neonates with clinical sign of neonatal sepsis and isolation of *C. parapsilosis* in the bloodstream and corresponded to group 1 of 17 patients. Controls were all neonates without *C. parapsilosis* isolation who were in the NICU for >48 h during the study period and were divided in groups 2–10 of 75 patients each using the chronological order from April 2009 to April 2012. Information was obtained retrospectively from patients’ notes, and characteristics and variables that were examined as possible risk factors are listed in Table 1. Length of NICU stay was calculated using the following formula: [(date of discharge from the NICU — date of admission to the NICU) + 1].

**Table 1:** Clinical and epidemiological features of *Candida parapsilosis* isolates in the neonatal intensive care unit

Patient code	Gender	Birth weight (g)	Gestational age (weeks)	Days of NICU stay	Days of NICU stay before isolation	Days of AV	Days of AV before isolation	Days of UC	Days of UC before isolation	Days of CVC	Days of CVC before isolation	Infection	Outcome	PCR fingerprinting genotype
OM	M	450	24	18	15	16	15	7	7	8	8	CVC-BSI	Death	A
G3D	M	560	24	26	14	26	14	11	11	15	3	UC-BSI	Death	A
AG	M	570	24	11	10	8	8	6	6	0	0	UC-BSI	Death	A
PR	M	580	25	5	5	5	5	5	5	0	0	UC-BSI	Death	A
BN	M	600	30	45	33	40	33	4	4	34	28	CVC-BSI	Death	A
PG	M	620	22	27	11	20	3	10	10	14	1	CVC-BSI	Death	A
TW	M	630	26	114	10	87	10	10	10	29	0	UC-BSI	Discharge	A
G2A	F	670	24	14	10	14	10	11	10	2	0	UC-BSI	Death	A
RD	M	790	27	234	5	108	4	3	3	15	2	UC-BSI	Discharge	A
DLBS	M	800	25	50	25	24	23	4	4	15	15	CVC-BSI	Death	A
MC	M	830	26	48	28	47	28	7	7	39	20	CVC-BSI	Death	A
OS	F	1160	32	31	18	0	0	3	3	22	14	CVC-BSI	Discharge	A
IC	F	1300	32	135	11	82	11	0	0	32	11	CVC-BSI	Discharge	A
DGR	M	1460	31	134	75	44	29	6	6	105	51	CVC-BSI	Discharge	A
SR	M	1700	32	70	18	5	5	2	2	67	16	CVC-BSI	Discharge	A
RE	M	2800	33	86	22	23	12	6	6	37	15	CVC-BSI	Discharge	A
RS	F	4280	40	76	18	47	18	4	4	38	18	CVC-BSI	Death	A

*UC* umbilical catheter, *CVC* central venous catheter, *AV* assisted ventilation, *UC- BSI* umbilical catheter-associated bloodstream infection, *CVC- BSI* central venous catheter-associated bloodstream infection

### Statistical analysis

Data were analyzed using R Statistical Package [26]. Risk factors for acquisition of *C. parapsilosis* were identified by a robust version of one-way independent ANOVA and a non-parametric counterpart as the Kruskal-Wallis test [27, 28]. For these univariate analyses, a *p*-value ≤0.05 was considered to indicate significance. Robust *Post hoc* tests for multiple pairwise case-control comparisons were run using Rand Wilcox’s functions [27, 28]. A Robust MANOVA was conducted on the ranked data using Munzel and Brunner’s [29] method, implemented in R using the mulrank function [27]. Variables tested for inclusion in the multi-variate model were those significantly associated with acquisition of *C. parapsilosis* at *p* ≤0.05 in the univariate analysis. A significant MANOVA was followed up using Discriminant Function Analysis (DFA) [30].

## Results

### Epidemiology of *C. parapsilosis* in NICU

Between April 2009 to April 2012 *C. parapsilosis* sepsis was diagnosed in 17 neonates, 13 males and 4 females, in the NICU. All 17 neonates with *C. parapsilosis* sepsis showed blood culture isolation (Table 1). Sixteen of the infected patients were pre-term neonates (median gestational age 26 weeks, IQR 24 – 32) and 11 of them were of extremely low birth weight (ELBW). The median birth weight was 790 g (IQR 600 – 1300). The median length of NICU stay was 48 days (IQR 26 – 86). During their NICU stay, 94.11% patients had an umbilical catheter (UC), 88.23% had a central venous catheter (CVC) and 94.11% underwent assisted ventilation (AV). The median length of exposure to a UC was 6 days (IQR 4 – 7), the median length of exposure to CVC was 22 days (IQR 14 – 37), and the median length of exposure to AV was 24 days (IQR 14 – 47). Six bloodstream infections were associated with the use of UC, 11 with the use of CVC. Fifty eight percent of neonates with *C. parapsilosis* bloodstream infection died (Table 1).

One of the 17 neonates with *C. parapsilosis* bloodstream infection showed concomitant isolation from pharynx surveillance swab. No additional pharynx or rectal swabs from the other 16 neonates were found positive for *C. parapsilosis*. During the study period, concomitant isolation of *Candida albicans* from pharynx or rectal surveillance swabs and emocolture was observed in 8 patients (data not shown). *C. parapsilosis* was isolated from the wall of neonatal intensive care room on July 2009. No *C. parapsilosis* isolates were obtained from staff hands. All *C. parapsilosis* isolates were identified to the species level and assigned to *C. parapsilosis* group I by PCR amplification and analysis of the internal transcribed spacer ribosomal DNA sequences. Molecular typing of *C. parapsilosis* isolates identified identical PCR fingerprinting profiles in *C. parapsilosis* isolates from the 17 neonates, blood culture and pharynx swab isolates from the same patient having identical fingerprinting profile also. In contrast, *C. parapsilosis* environmental isolate showed different PCR fingerprinting profiles (Additional file 1 Table S1). The above finding excluded the contaminated environment of the NICU as the source or reservoir of *C. parapsilosis* in the ward and demonstrated the cross-transmission of one single *C. parapsilosis* clone among neonates.

### Antimycotic susceptibility profiles of *C. parapsilosis* isolates

All *C. parapsilosis* isolates were susceptible to anidulafungin, micafungin, caspofungin, 5-fluorocytosine, amphotericin B, posaconazole and voriconazole. Fluconazole resistance was detected in two isolates (MICs 128 *μ*g/mL and 64 *μ*g/mL, respectively), intermediate susceptibility to itraconazole (MIC 0.25 *μ*g/mL) was detected in one of the above two isolates (Table 2). Both fluconazole-resistant *C. parapsilosis* isolates were from neonates ≤1500 g BW.

**Table 2:** Antimycotic susceptibility profiles of *C. parapsilosis* isolates

Antifungal Agents	MIC_50_ (*μ*g/mL)	MIC_90_ (*μ*g/mL)	MIC range (*μ*g/mL)
Anidulafungin	1	2	0.5–2
Micafungin	2	2	1–2
Caspofungin	0.5	1	0.25–2
5-fluorocytosine	0.25	0.5	0.12–0.5
Posaconzole	0.06	0.125	0.03–0,125
Voriconazole	0.03	0.5	0.008–1
Itraconazole	0.12	0.25	0.06–0,25
Fluconazole	1	64	0.5–128
Amphotericin B	0.5	1	0.5–1

*MIC* minimal inhibitory concentration

### Risk factor analysis

**Fig. 1:**
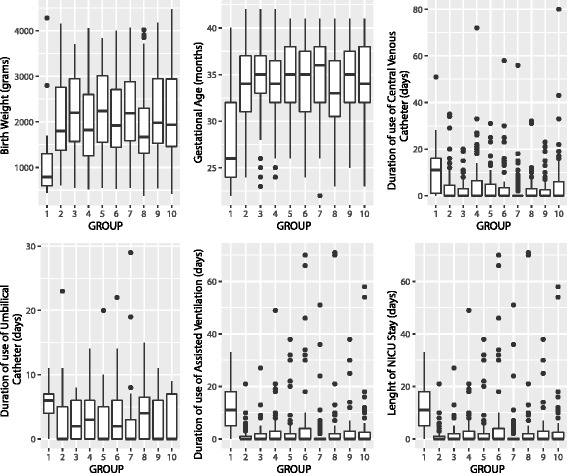
Boxplots for covariates counts in each group. Group 1 represents the cases, groups 2–10 represent the controls. The circles that lie above the top whiskers are the outliers

**Fig. 2:**
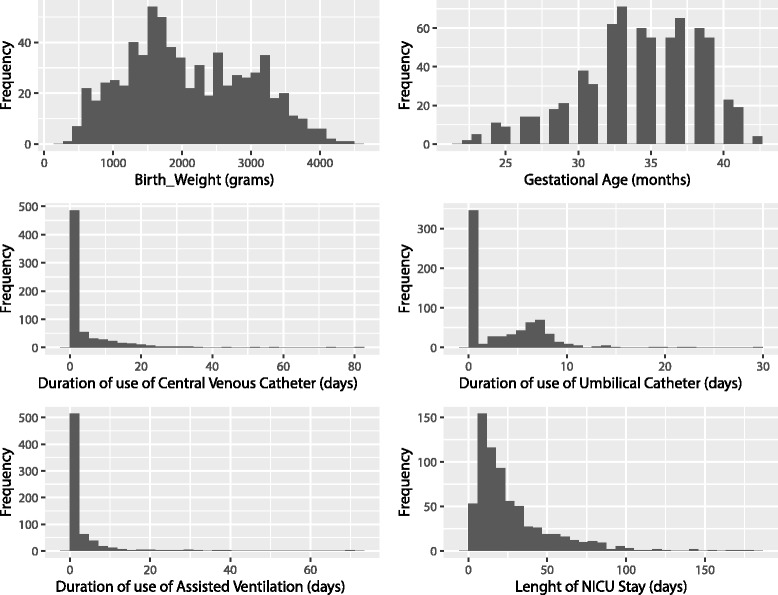
Histograms of variable distributions. Sampling distributions are not normally distributed

Figure 1 showed the boxplots for covariates counts in cases and control groups, whereas Fig. 2 showed that the overall sampling distributions were not normally distributed. Moreover, in the case of duration of use of CVC and duration of use of AV, the variances in different groups were different as stated by the Levene’s Test for Homogeneity of Variance (*p* <0.001). Instead, duration of use of umbilical catheter, gestational age, birth weight, and length of NICU stay, showed homogeneity of variance in different groups. Moreover, except for the cases of gestational age, birth weight, and length of NICU stay, we cannot assume normality of residuals errors. For these reasons, both a Robust ANOVA method, based on bootstrapping and/or trimmed means and M-estimators, and a non-parametric method, such as Kruskal-Wallis were used for risk factor analysis. Additional file 1 Table S2 showed also *Post hoc* tests, i.e. pairwise comparisons that were designed to compare all different combinations of the treatment groups, for both methods. Univariate analysis showed that *C. parapsilosis* bloodstream infection was significantly related to gestational age, birth weight, duration of use of UC, CVC and AV, but not to length of NICU stay.

MANOVA multivariate analysis was performed to detect the relationship among outcome variables and the differences of groups along a combination of dimensions. Robust MANOVA results showed a significant effect for acquisition of *C. parapsilosis:* F = 3.043, *p* <0.001. Moreover, the rank values of duration of use of AV (.72), Gestational Age (.75), and Birth Weight (.88), were greater in cases (group 1) than controls (groups 2–10) (Table 3). The reverse was true for duration of use of CVC (.17), and duration of use of Umbilical Catheter (.19): the ranks of the cases were lower than those of the controls (Table 3). Next, discriminant function analysis was performed to distinguish a set of groups using several predictors. The ratio of systematic to unsystematic variance was maximized for this first group, with subsequent groups having smaller values, as shown by the trend of proportion of trace in Table 4. Hence, the coefficients measured the relative contribution of each variable to the discrimination among the groups. Noteworthy, gestational age and birth weight were highly correlated with rho = 0.841 (Spearman’s rank test *p*-value <0.01), thus resulting as a single predictor in DFA. Discriminant analysis revealed five discriminant functions (Table 4). The proportion of trace showed that the first group (practically gestational age) accounted for 54.5% of variance compared to the second group (gestational age plus duration of use of UC and duration of use of AV), which accounted for 22.2%. The third group (gestational age plus duration of use of AV) accounted for 12.3%, while the fourth and the fifth groups accounted for 7.1%, and 4.0%, respectively.

**Table 3:** Relative effect of Robust MANOVA

Group	Duration of use of CVC	Duration of use of UC	Duration of use of AV	Gestational Age	Birth Weight
1	0.167	0.186	0.720	0.745	0.877
2	0.479	0.394	0.531	0.494	0.490
3	0.395	0.384	0.559	0.540	0.488
4	0.555	0.548	0.567	0.608	0.531
5	0.501	0.514	0.428	0.575	0.462
6	0.515	0.527	0.483	0.488	0.534
7	0.443	0.424	0.539	0.428	0.523
8	0.549	0.518	0.471	0.426	0.409
9	0.552	0.568	0.426	0.424	0.450
10	0.543	0.547	0.464	0.504	0.559

**Table 4:** Discriminant Function Analysis

	LD1	LD2	LD3	LD4	LD5
Duration of use of CVC	−0.033	−0.102	0.058	0.076	0.007
Duration of use of UC	0.002	0.105	−0.118	0.227	−0.147
Duration of use of AV	−0.033	0.111	0.111	−0.042	0.025
Gestational Age	0.228	0.149	0.149	0.247	0.262
Birth Weight	−0.0002	−0.0003	−0.0003	−0.0005	−0.002
Proportion of trace	0.545	0.222	0.123	0.071	0.040

## Discussion

*C. parapsilosis* is an increasing cause of healthcare-associated infections and in particular device-associated blood stream infections in the NICU [6, 11–13, 31]. In the tertiary care level NICU of University Hospital in Naples, Italy, *C. parapsilosis* was responsible for 50% and 35% of UC-associated and CVC-associated bloodstream infections during 2006–2010 [6].

The data presented here demonstrate that a single clone of *C. parapsilosis* was responsible for UC or CVC-associated bloodstream infections in 17 neonates from our NICU from April 2009 to April 2012. *C. parapsilosis* strains assigned to *C. parapsilosis* group I and showing identical PCR fingerprinting profiles were isolated from blood cultures of all 17 neonates and pharynx swab of 1 neonate, while *C. parapsilosis* environmental isolate showed different PCR fingerprinting profiles. A previous study showed that that the increase of *C. parapsilosis* bloodstream infections in a NICU in Helsinki, Finland was caused by the spread of a single clone with identical DNA fingerprinting profile [14]. In accordance with previous findings [13, 14], the majority (15 out of 17) *C. parapsilosis* isolates were susceptible to echinocandins, azoles, 5-fluorocytosine and amphotericin B, but 2 were resistant to fluconazole and one resistant to fluconazole and intermediate resistant to itraconazole. The occurrence of resistance to fluconazole in *C. parapsilosis* isolates from 2 out of 14 neonates ≤1500 g BW under fluconazole prophylaxis is in agreement with previous study showing that fluconazole prophylaxis increase the incidence of invasive infections involving fluconazole-resistant *C. parapsilosis* [31].

Molecular typing data presented herein exclude the environment as the source or reservoir of *C. parapsilosis* in the ward. Also, the absence of isolation of *C. parapsilosis* from the pharynx or rectal swabs in 16 neonates with *C. parapsilosis* bloodstream infection and the concomitant isolation of *C. parapsilosis* from pharynx swab and emocolture in only 1 neonate suggest that mucosal colonization by *C. parapsilosis* does not precede invasive infection as observed for *C. albicans* bloodstream infection. Although our data did not identify the route of transmission of *C. parapsilosis* among neonates in the NICU, the assignment of all *C. parapsilosis* clinical isolates to a single epidemic genotype suggests that infected neonates were the reservoir and source of *C. parapsilosis* epidemic in the NICU.

Several studies analyzed risk factors for neonatal candidiasis without distinguishing among isolated *Candida* species [7–11], while few studies analyzed risk factors for invasive *C. parapsilosis* infections in neonates by univariate analysis [13–15] or multivariate analysis [15–17]. Neonatal risk factors for invasive *C. parapsilosis* infections were birth weight <1500 g, prematurity, prior colonization, parenteral nutrition, intravascular catheters and use of antibiotics, steroids and H2 blockers (13–15). Similarly, we found that birth weight, gestational age, time to exposure to UC, CVC, and AV were risk factors for *C. parapsilosis* infection in neonates in the NICU at univariate analysis. In a case-control population-based study on 78 episodes of *C. parapsilosis* fungemia compared with 175 *C. albicans* controls, neonate patients, transplant patients, and patients who received antifungal therapy or parenteral nutrition were significantly associated with *C. parapsilosis* bloodstream infection on multivariate analysis [15]. When non-*albicans Candida* species bloodstream infections including those caused by *C. parapsilosis* were compared with *C. albicans* bloodstream infections, multivariate logistic regression analysis identified neonatal age, fluconazole exposure and having received an hematological transplant as factors associated with a risk of candidemia caused by non-*albicans Candida* species [16]. Risk factors analysis of nosocomial candidemia in an adult intensive care unit (ICU) and a NICU in Brazil identified sex and parenteral nutrition as independent risk factors of *C. parapsilosis* bloodstream infections, which were more frequent in NICU than in adult ICU [17]. In agreement with these findings, we identified low birth weight and gestational age (explaining more than 55.0% of the variance) as risk factors for *C. parapsilosis* bloodstream infection in neonates in the NICU. Moreover, we demonstrated that time to exposure to invasive devices (explaining ca 34.0% of the variance), with predominance of assisted ventilation, is independent risk for *C. parapsilosis* bloodstream infection in neonates in the NICU at multivariate analysis. Based on our data, we speculate that prematurity and extremely low birth weight (<1000 g) and any manoeuvre associated with AV usage may have been involved in the transmission between patients and acquisition of *C. parapsilosis* infections in our NICU.

We recognize that our study has limitations that affect the generalization of our results. The first limitation relies on the retrospective nature of the study, which did not allow to evaluate the efficacy of specific infection prevention measures against *C. parapsilosis* bloodstream infection in the NICU. For example, the appropriateness of antifungal prophylaxis was not assessed by our study despite the isolation of two *C. parapsilosis* isolates resistant to fluconazole. Additional limitation of the study was the lack of analysis of inborn and outborn status, mortality, total parenteral nutrition, probiotics administration, days and type of antenatal and postnatal antibiotic use and other concomitant drugs therapeutic variables of neonates included in the study. Future studies will be necessary to investigate the above issues.

## Conclusion

*C. parapsilosis* UC or CVC-associated bloodstream infections in our NICU were caused by the spread of a single epidemic clone. Low birth weight, gestational age, duration of use of UC, duration of use of CVC, duration of use of AV were risk factors for *C. parapsilosis* infection in neonates in the NICU at univariate analysis. Low birth weight, gestational age and time to exposure to AV were identified as principal independent risk factors for *C. parapsilosis* bloodstream at multivariate analysis. Surveillance of device-associated infections are necessary to prevent *C. parapsilosis* bloodstream infections in the NICU.

## Additional file

Additional file 1: Table S1. PCR fingerprinting profiles of *C. parapsilosis* isolates. Table S2. Robust ANOVA and Kruskal-Wallis tests. (PDF 71 kb)

## Abbreviations

AV: Assisted ventilation
BSI: Bloodstream infection
BW: Birth weight
CVC: Central venous catheter
DFA: Discriminant function analysis
ELBW: Extremely low birth weight
HAIs: Healthcare-associated infections
MIC: Minimal inhibitory concentration
NICU: Neonatal intensive care
UC: Umbilical catheter

## References

[1] Polin RA, Denson S, Brady MT (2012). Committee on fetus and newborn; committee on infectious diseases. Epidemiology and diagnosis of health care-associated infections in the NICU. Pediatrics.

[2] Hooven TA, Polin RA (2014). Healthcare-associated infections in the hospitalized neonate: a review. Early Hum Dev.

[3] Geffers C, Baerwolff S, Schwab F, Gastmeier P (2008). Incidence of healthcare-associated infections in high-risk neonates: results from the German surveillance system for very-low-birthweight infants. J Hosp Infect.

[4] Verstraete E, Boelens J, De Coen K, Claeys G, Vogelaers D, Vanhaesebrouck P, Blot S (2014). Healthcare-associated bloodstream infections in a neonatal intensive care unit over a 20-year period (1992–2011): trends in incidence, pathogens, and mortality. Infect Control Hosp Epidemiol.

[5] Auriti C, Ronchetti MP, Pezzotti P, Marrocco G, Quondamcarlo A, Seganti G, Bagnoli F, De Felice C, Buonocore G, Arioni C (2010). Determinants of nosocomial infection in 6 neonatal intensive care units: an Italian multicenter prospective cohort study. Infect Control Hosp Epidemiol.

[6] Crivaro V, Bogdanović L, Bagattini M, Iula VD, Catania M, Raimondi F, Triassi M, Zarrilli R (2015). Surveillance of healthcare-associated infections in a neonatal intensive care unit in Italy during 2006–2010. BMC Infect Dis.

[7] Benjamin DK, Stoll BJ, Gantz MG, Walsh MC, Sánchez PJ, Das A, Shankaran S, Higgins RD, Auten KJ, Miller NA (2010). Eunice Kennedy Shriver national institute of child health and human development neonatal research network. Neonatal candidiasis: epidemiology, risk factors, and clinical judgment. Pediatrics.

[8] Kelly MS, Benjamin DK, Smith PB (2015). The epidemiology and diagnosis of invasive candidiasis among premature infants. Clin Perinatol.

[9] Barton M, O’Brien K, Robinson JL, Davies DH, Simpson K, Asztalos E, Langley JM, Le Saux N, Sauve R, Synnes A (2014). Invasive candidiasis in low birth weight preterm infants: risk factors, clinical course and outcome in a prospective multicenter study of cases and their matched controls. BMC Infect Dis.

[10] Yu Y, Du L, Yuan T, Zheng J, Chen A, Chen L, Shi L (2013). Risk factors and clinical analysis for invasive fungal infection in neonatal intensive care unit patients. Am J Perinatol.

[11] Pasqualotto AC, de Moraes AB, Zanini RR, Severo LC (2007). Analysis of independent risk factors for death among pediatric patients with candidemia and a central venous catheter in place. Infect Control Hosp Epidemiol.

[12] Guinea J (2014). Global trends in the distribution of *Candida* species causing candidemia. Clin Microbiol Infect.

[13] Pammi M, Holland L, Butler G, Gacser A, Bliss JM (2013). *Candida parapsilosis* is a significant neonatal pathogen: a systematic review and meta-analysis. Pediatr Infect Dis J.

[14] Sarvikivi E, Lyytikäinen O, Soll DR, Pujol C, Pfaller MA, Richardson M, Koukila-Kähkölä P, Luukkainen P, Saxén H (2005). Emergence of fluconazole resistance in a *Candida parapsilosis* strain that caused infections in a neonatal intensive care unit. J Clin Microbiol.

[15] Almirante B, Rodríguez D, Cuenca-Estrella M, Almela M, Sanchez F, Ayats J, Alonso-Tarres C, Rodriguez-Tudela JL, Pahissa A (2006). Epidemiology, risk factors, and prognosis of *Candida parapsilosis* bloodstream infections: case-control population-based surveillance study of patients in Barcelona, Spain, from 2002 to 2003. J Clin Microbiol.

[16] Rodríguez D, Almirante B, Cuenca-Estrella M, Rodríguez-Tudela JL, Mensa J, Ayats J, Sanchez F, Pahissa A (2010). Barcelona candidemia project study group. Predictors of candidaemia caused by non-albicans *Candida* species: results of a population-based surveillance in Barcelona, Spain. Clin Microbiol Infect.

[17] Hoffmann-Santos HD, Paula CR, Yamamoto AC, Tadano T, Hahn RC (2013). Six-year trend analysis of nosocomial candidemia and risk factors in two intensive care hospitals in Mato Grosso, midwest region of Brazil. Mycopathologia.

[18] Manzoni P, Stolfi I, Pugni L, Decembrino L, Magnani C, Vetrano G, Tridapalli E, Corona G, Giovannozzi C, Farina D (2007). Italian task force for the study and prevention of neonatal fungal infections; Italian society of neonatology. A multicenter, randomized trial of prophylactic fluconazole in preterm neonates. N Engl J Med.

[19] NNIS System (1999). National nosocomial infections surveillance (NNIS) system report, data summary from January 1990-May 1999, issued June 1999. A report from the NNIS system. Am J Infect Control.

[20] Jarvis WR (2003). Benchmarking for prevention: the centers for disease control and Prevention’s national nosocomial infections surveillance (NNIS) system experience. Infection.

[21] Horan TC, Andrus M, Dudeck MA (2008). CDC/NHSN surveillance definition of health care-associated infection and criteria for specific types of infections in the acute care setting. Am J Infect Control.

[22] Pulcrano G, Roscetto E, Iula VD, Panellis D, Rossano F, Catania MR (2012). MALDI-TOF mass spectrometry and microsatellite markers to evaluate *Candida parapsilosis* transmission in neonatal intensive care units. Eur J Clin Microbiol Infect Dis.

[23] Tavanti A, Davidson AD, Gow NA, Maiden MC, Odds FC (2005). *Candida orthopsilosis* and *Candida metapsilosis* spp. nov. to replace *Candida parapsilosis* groups II and III. J Clin Microbiol.

[24] Xu J, Ramos AR, Vilgalys R, Mitchell TG (2000). Clonal and spontaneous origins of fluconazole resistance in Candida albicans. J Clin Microbiol.

[25] Clinical and Laboratory Standards Institute (2012). Reference method for broth dilution antifungal susceptibility testing of yeasts; fourth informational supplement. CLSI document M27-S4.

[26] The R Project for Statistical Computing. https://www.r-project.org/. Accessed 06 May 2016.

[27] Wilcox RR (2005). Introduction to robust estimation and hypothesis testing.

[28] Field AP, Miles J, Field Z (2012). Discovering statistics using R.

[29] Munzel U, Brunner E (2000). Nonparametric tests in the unbalanced multivariate one-way design. Biom J.

[30] McLachlan GJ (2004). Discriminant analysis and statistical pattern recognition.

[31] Lee J, Kim HS, Shin SH, Choi CW, Kim EK, Choi EH, Kim BI, Choi JH (2016). Efficacy and safety of fluconazole prophylaxis in extremely low birth weight infants: multicenter pre-post cohort study. BMC Pediatr.

