# Supplementary material for: Risk factors for Candida parapsilosis bloodstream infection in a neonatal intensive care unit: a case-control study
Source: Ital J Pediatr. 2017 Jan 19;43:10. doi: 10.1186/s13052-017-0332-5 (PMC5347820; doi:10.1186/s13052-017-0332-5)
Supplement: Additional file 1: Table S1. — PCR fingerprinting profiles of C. parapsilosis isolates. Table S2. Robust ANOVA and Kruskal-Wallis tests. (PDF 71 kb) [file 13052_2017_332_MOESM1_ESM.pdf]

Table S1. PCR fingerprinting profiles of *C. parapsilosis* isolates

**Patient code**	**GACA4**										**M13**							**T3B**									**OPA-03**											
OM	1	0	1	1	1	1	1	0	0	A	0	1	1	1	0	1	A	1	0	0	0	0	1	1	0	A	1	1	1	1	1	1	1	1	1	1	1	A
G3D	1	0	1	1	1	1	1	0	0	A	0	1	1	1	0	1	A	1	0	0	0	0	1	1	0	A	1	1	1	1	1	1	1	1	1	1	1	A
AG	1	0	1	1	1	1	1	0	0	A	0	1	1	1	0	1	A	1	0	0	0	0	1	1	0	A	1	1	1	1	1	1	1	1	1	1	1	A
PR	1	0	1	1	1	1	1	0	0	A	0	1	1	1	0	1	A	1	0	0	0	0	1	1	0	A	1	1	1	1	1	1	1	1	1	1	1	A
BN	1	0	1	1	1	1	1	0	0	A	0	1	1	1	0	1	A	1	0	0	0	0	1	1	0	A	1	1	1	1	1	1	1	1	1	1	1	A
PG	1	0	1	1	1	1	1	0	0	A	0	1	1	1	0	1	A	1	0	0	0	0	1	1	0	A	1	1	1	1	1	1	1	1	1	1	1	A
TW	1	0	1	1	1	1	1	0	0	A	0	1	1	1	0	1	A	1	0	0	0	0	1	1	0	A	1	1	1	1	1	1	1	1	1	1	1	A
G2A	1	0	1	1	1	1	1	0	0	A	0	1	1	1	0	1	A	1	0	0	0	0	1	1	0	A	1	1	1	1	1	1	1	1	1	1	1	A
RD	1	0	1	1	1	1	1	0	0	A	0	1	1	1	0	1	A	1	0	0	0	0	1	1	0	A	1	1	1	1	1	1	1	1	1	1	1	A
DLBS	1	0	1	1	1	1	1	0	0	A	0	1	1	1	0	1	A	1	0	0	0	0	1	1	0	A	1	1	1	1	1	1	1	1	1	1	1	A
MC	1	0	1	1	1	1	1	0	0	A	0	1	1	1	0	1	A	1	0	0	0	0	1	1	0	A	1	1	1	1	1	1	1	1	1	1	1	A
OS	1	0	1	1	1	1	1	0	0	A	0	1	1	1	0	1	A	1	0	0	0	0	1	1	0	A	1	1	1	1	1	1	1	1	1	1	1	A
IC	1	0	1	1	1	1	1	0	0	A	0	1	1	1	0	1	A	1	0	0	0	0	1	1	0	A	1	1	1	1	1	1	1	1	1	1	1	A
DGR	1	0	1	1	1	1	1	0	0	A	0	1	1	1	0	1	A	1	0	0	0	0	1	1	0	A	1	1	1	1	1	1	1	1	1	1	1	A
SR	1	0	1	1	1	1	1	0	0	A	0	1	1	1	0	1	A	1	0	0	0	0	1	1	0	A	1	1	1	1	1	1	1	1	1	1	1	A
RE	1	0	1	1	1	1	1	0	0	A	0	1	1	1	0	1	A	1	0	0	0	0	1	1	0	A	1	1	1	1	1	1	1	1	1	1	1	A
RS	1	0	1	1	1	1	1	0	0	A	0	1	1	1	0	1	A	1	0	0	0	0	1	1	0	A	1	1	1	1	1	1	1	1	1	1	1	A
environmental sample	1	1	1	1	0	1	1	1	1	B	1	0	0	1	1	1	B	0	1	0	1	1	0	0	1	B	1	1	1	0	0	1	0	1	1	1	1	B

PCR primers used to generate fingerprinting profiles are indicated. Numbers 1 and 0 indicate the presence or the absence of the PCR product, respectively.

Table S2. Robust ANOVA and Kruskal-Wallis tests

	Robust ANOVA p	Multiple Case-Control comparison test after Robust ANOVA p*	Kruskal-Wallis rank sum test p	Multiple Case-Control comparison test after Kruskal-Wallis p*
Duration of Use of CVC	0.0291	< 0.05	0.0298	< 0.05
Duration of Use of UC	<0.001	< 0.05	<0.001	< 0.05
Duration of Use of AV	<0.001	< 0.05	<0.001	< 0.05
Gestational Age	<0.001	< 0.05	<0.001	< 0.05
Birth Weight	<0.001	< 0.05	<0.001	< 0.05
Length of NICU Stay	NS	NS	NS	NS

*For all pairwise comparisons between groups.

Notes. For Robust ANOVA trimming was 20%. A bootstrap with n=5000 bootstrap samples was added to the trimmed mean.

Abbreviations: UC, umbilical catheter; CVC, central venous catheter; AV, assisted ventilation; NS, not significant.
